# Attract to kill: Exploring the potential of motility trapping as a novel treatment strategy for high-grade gliomas

**DOI:** 10.1093/neuonc/noag067

**Published:** 2026-03-27

**Authors:** Anne-Sophie Bamelis, Dennis Serge Metselaar, Mariska Sie, Thibault Lootens, Leen Willems, Robrecht Raedt, Jelle Vandersteene

**Affiliations:** Department of Neurosurgery, Ghent University Hospital, Ghent, Belgium; 4Brain Lab, Department of Head and Skin, Ghent University, Ghent, Belgium; Cancer Research Institute Ghent (CRIG), Ghent, Belgium; Princess Máxima Center for Pediatric Oncology, Utrecht, The Netherlands; Hopp Children’s Cancer Center Heidelberg (KiTZ), Heidelberg, Germany; Division of Pediatric Neurooncology, German Cancer Research Center (DKFZ) and German Consortium (DKTK), Heidelberg, Germany; Princess Máxima Center for Pediatric Oncology, Utrecht, The Netherlands; 4Brain Lab, Department of Head and Skin, Ghent University, Ghent, Belgium; Cancer Research Institute Ghent (CRIG), Ghent, Belgium; Laboratory of Experimental Cancer Research (LECR), Department of Human Structure and Repair, Ghent University, Ghent, Belgium; Department Pediatric Hematology and Oncology, Ghent University Hospital, Ghent, Belgium; 4Brain Lab, Department of Head and Skin, Ghent University, Ghent, Belgium; Cancer Research Institute Ghent (CRIG), Ghent, Belgium; Department of Neurosurgery, Ghent University Hospital, Ghent, Belgium; 4Brain Lab, Department of Head and Skin, Ghent University, Ghent, Belgium; Cancer Research Institute Ghent (CRIG), Ghent, Belgium

## Abstract

High-grade gliomas (HGG) are notoriously hard to treat due to surgical limitations and resistance to systemic therapies, resulting in a dire prognosis. Tumor cell motility is a major contributor to treatment failure but simultaneously offers a therapeutic opportunity, utilizing a novel approach called “motility trapping”. Motility trapping leverages chemotactic signals to redirect tumor cells to a location where local therapies can target them more effectively. This concept can be applied *inward*, drawing disseminated tumor cells back to the primary tumor, or *outward*, guiding them toward a more therapy-accessible location. While preclinical research demonstrates that *motility trapping* can influence tumor migration, clinical translation remains unestablished. To advance clinical applicability, 4 essential components must be considered: effective migratory stimuli, susceptible tumor cells, suitable delivery systems, and the influence of the tumor microenvironment. For each element, we review current knowledge and propose future directions to develop this innovative approach. In conclusion, redirecting HGG migration through motility trapping offers a transformative strategy that warrants further preclinical and translational investigation. It holds promise to synergize with a plethora of therapeutic strategies that are currently ineffective in brain tumors and should be considered in the design of future therapies.

Key Points

- Motility trapping, attracting tumor cells to sites amenable to therapy, has proven feasible preclinically.
- As it synergizes with local, immune, and other systemic therapies, it holds potential to improve survival and minimize side effects.
- This review provides a roadmap toward clinical translation.

High-grade gliomas (HGGs) are among the greatest unmet needs in neuro-oncology. They encompass multiple subtypes across pediatric and adult patients, all characterized by aggressive behavior and a dismal prognosis. Glioblastoma (GBM), the most common subtype, affects 322 out of 100,000 people and carries a median overall survival of 14-16 months.^1^ Diffuse midline glioma (DMG) is less prevalent, primarily affects children, and has a median overall survival of only <1 year after diagnosis.^2^^,^^3^

These poor outcomes persist despite aggressive multimodal treatment, highlighting limitations of both systemic and local therapies. Systemic drug delivery is hindered by the blood-brain barrier (BBB), making local therapy, including surgical resection and radiotherapy, the cornerstone of treatment.^4^ However, local treatment is often hindered by anatomical constraints and, in some pediatric cases, age limitations. Moreover, HGG cells typically disseminate outside the visible tumor margins and into healthy brain tissue, thereby evading local therapy.^5^ Tumor cell motility is thus a key factor of therapy failure and tumor progression.

Tumor cell motility combines local migration into adjacent structures and distant migration to remote brain locations.^6^ Local migration is often considered the predominant cause of recurrence, which most frequently arises near the original tumor site.^7^ However, previous efforts to increase the extent of resection and thus eliminate locally infiltrated tumor cells only modestly improved overall survival and failed to prevent tumor recurrence.^8^ This suggests that local recurrence may also result from distant migration, as shown in a preclinical mouse model where GBM recurrence at the resection site resulted from tumor cells from the subventricular zone (SVZ).^9^ Consequently, treatment strategies targeting tumor cell motility should address both local and distant migration.

Historically, research mainly focused on inducing cancer cell death, but recent studies have increased our understanding of tumor migration and the influence of the complex tumor microenvironment (TME). Moreover, targeting tumors by modulating the TME has shown promise in HGG, exemplified by the success of recent CAR-T therapies that direct immune cells to the TME.^10^ Motility trapping builds on this concept by leveraging migratory signals from the TME, for example, soluble proteins known as chemoattractants, as bait to attract migratory tumor cells.

The potential of motility trapping has been explored in various extracranial solid tumors.^11^ Van der Sanden et al^12^ first proposed motility trapping as a conceptual treatment strategy for primary brain tumors, but knowledge gaps prevented its clinical development. Our review expands the concept of motility trapping in light of recent advances in tumor biology and the interaction with the TME. We propose HGG as ideal candidates for this strategy due to their highly infiltrative nature, reliance on local therapy, and urgent need for innovative treatments. This paper explores the potential of motility trapping in HGG and aims to guide its future development.

## Principle of Motility Trapping

Motility trapping is based on the ecological trap principle, where migratory cues mislead an organism to an unfavorable location, resulting in its elimination. Translated to oncology, this concept becomes a 2-step approach. First, tumor cells are redirected by synthetically administering the migratory stimulus, for example, the chemoattractant. Second, the attracted tumor cells are eradicated by local therapy. This converts the high degree of tumor cell motility in HGG into a therapeutic opportunity. In short, the tumor is susceptible to being killed.

**Figure 1.**
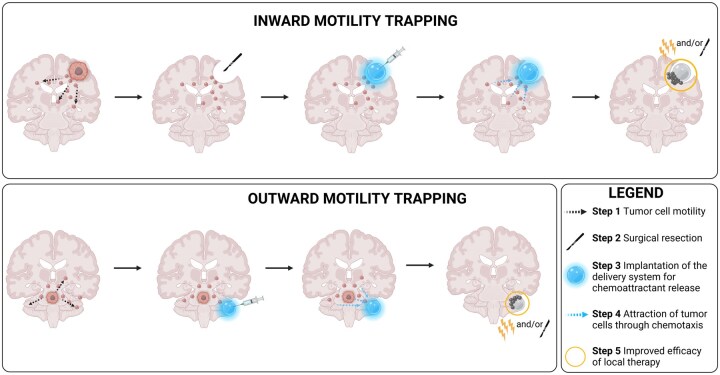
Conceptual overview of the two motility trapping strategies. An inward motility trap attracts disseminated tumor cells back to the original tumor location (eg, after resection) to be eliminated by subsequent local therapy. Conversely, outward motility trapping redirects tumor cells to an extratumoral location that is more accessible to local therapy. Both strategies aim to enhance the efficacy of local therapy (Created in BioRender. Bamelis, A. (2026) https://BioRender.com/1t85bks).

Depending on tumor characteristics, 2 strategies for motility trapping emerge (Figure 1). The first, *inward motility trapping*, involves the administration of the migratory stimulus at the primary tumor location, thereby reversing tumor dissemination. *Outward motility trapping* places the stimulus at a distance from the primary tumor to redirect migratory tumor cells to a location of choice.

Inward motility trapping aims to attract invasive tumor cells back toward the primary tumor location, potentially offering several benefits. Most importantly, it increases the number of tumor cells targeted by local therapy and decreases residual tumor burden. This may be comparable to extended surgical resection, which has been shown to increase overall survival.^13^ Preventing further migration into the surrounding brain tissue or leptomeningeal spread may slow disease progression and delay neurological decline. Additionally, in tumors with a disrupted BBB—typically the case in GBM, particularly after resection^14^—inward motility trapping concentrates more tumor cells into regions where the BBB is compromised and increases exposure to systemic treatment.

In tumors where inward motility trapping is anatomically challenging and local therapy less favorable (eg, eloquent areas, particularly deep midline structures or the brainstem), outward motility trapping may offer greater benefit as it shifts the tumor burden from critical areas to locations more amenable to treatment. This could increase the fraction of tumor cells within reach of local therapies while reducing the risk of functional damage from local therapy to the surrounding critical tissue. For example, pontine DMG can cause increased intracranial pressure (ICP) due to tumor growth within this confined anatomical region. Redirecting tumor cells elsewhere using an outward motility trap might reduce tumor burden in the pons and potentially alleviate this mass effect. Additionally, local therapies often induce edema around the treatment site. Outward trapping may avoid exacerbating swelling in already restricted spaces, minimizing further neurological risk.

Understanding the principle of motility trapping lays the foundation for further examination of this approach. The next step involves the identification of the key components required for successful tumor attraction and eradication, as discussed in the next chapter.

## Essential Components to Achieve Effective Motility Trapping

We propose 4 elements to consider in the development of a motility trap: an effective migratory stimulus, susceptible tumor cells, a suitable delivery system, and the influence of the TME.

### The Search for an Effective Migratory Stimulus

Effective motility trapping depends on a migratory stimulus to induce directional tumor migration. In HGG, tumor cell motility is guided by the TME through chemical or electrical signaling.

#### Chemotaxis

Motility traps are typically composed of chemotactic cytokines, referred to as chemokines.^11^ These small signaling molecules have an important role in immune surveillance and inflammation throughout the body but are also essential in the central nervous system. In the healthy brain, chemokines regulate critical processes, for example, maintenance and migration of neural stem cells (NSCs).^15^ Their concentration is unevenly distributed in the brain, with the highest level found in the basal ganglia and limbic system and this underlines their capacity to form physiological gradients necessary to direct cell movement.^16^ HGG hijacks this signaling system to drive its migratory behavior. Within the TME, chemokines are secreted by glial cells, endothelial cells, neurons, and tumor cells themselves.^17-19^ Extracellular gradients are sensed by HGG cells through surface receptors. Binding of chemokines activates various intracellular pathways that promote migration, for example, by inducing a mesenchymal shift.

An overview of chemokines that have proven to influence HGG migration and their corresponding receptors is given in Table 1. The most studied chemokine in this context is CXCL12, or stromal-derived factor 1, which has been proven to attract HGG cells under *in vitro* conditions.^20^^,^^21^ When CXCL12 binds to its receptor CXCR4, multiple downstream pathways are activated, for example, the ERK1/2 and Akt pathway, promoting tumor cell motility through upregulation of matrix-metalloproteinases (MMP) and cytoskeletal reorganization. The functional relevance of this axis is further supported by *in vivo* models. Glioma stem cells migrate from the primary tumor toward the SVZ, following a CXCL12 gradient, in U87-MG- and GB138-xenografted mice.^22^ Interestingly, daily treatment of these mice with a CXCR4 antagonist blocked this SVZ-specific migration. While CXCL12 overexpression in the human SVZ has not been proven, it is abundant at the invasive edge of GBM, creating a potent chemotactic gradient.^23^ CXCR4 is also frequently expressed by HGG cells, enabling them to respond to this gradient.^24^ Besides CXCL12, various other chemokines have been demonstrated to induce directional migration in HGG in preclinical research, with, for example, CCL2, CXCL10, and CCL11 inducing both inward and outward motility trapping *in vivo*.^25^ Advances in sequencing techniques could identify additional chemotactic candidates in HGG. Receptors selectively overexpressed in tumor cells with downstream effects on migration should be considered as a starting point. Next, migratory responses should be tested *in vitro* and, if confirmed, validated *in vivo*.

**Table 1.** Overview of chemokines and the corresponding receptors, alongside their role in HGG cell motility

Chemokine (alternative name)	Receptor(s)	Evidence for their role in HGG cell motility
CCL2 (MCP-1)	CCR2, CCR4	Overexpression of CCL2 in U87-MG cells enhances migration *in vitro*, and this effect is amplified in co-culture with CCR2-expressing microglia.^92^ An *in vitro* CCL2 gradient from an agarose drop increases the number of migrated cells and distance traveled of U87-MG and F98 cells, compared to PBS-loaded drops.^25^ Both inward and outward motility trapping using CCL2 is proven in the F98 orthotopic rat model.^25^
CCL5 (RANTES)	CCR5	CCL5 stimulates migration of U87-MG and U251 cells in an *in vitro* Transwell assay.^93^
CCL8 (MCP-2)	CCR1, CCR3, CCR5	CCL8 in the TME promotes the formation of pseudopodia, an important hallmark for invasiveness, in 3D cell culture of 2 primary GBM cell lines.^94^ Co-implantation of GL-261 cells and CCL8 enhances migration and decreases survival in immunodeficient mice, in comparison to inoculation of tumor cells alone.^94^
CCL11 (Eotaxin-1)	CCR3	Both a neutralizing CCL11 antibody and an inhibitory short hairpin RNA to silence CCR3 decrease migration of U251 and U87-MG cells in 2 separate *in vitro* assays, a wound healing and a Transwell assay, highlighting the role of CCL11 in tumor motility.^95^ In the agarose drop assay, CCL11 increases U87-MG migration but has no effect on F98 cells. In the rat F98 model, an intratumoral CCL11 hydrogel reduces the number of peritumoral clusters as a surrogate for tumor migration, while contralateral implantation increased it compared to non-loaded hydrogels.^25^
CXCL1 (Gro-α/MGSA-α)	CXCR2	U251 cells transfected with viral vector to overexpress CXCL1 have increased motility in *in vitro* and in a U251-xenograft mice model.^96^
CXCL7 (NAP-2)	CXCR1, CXCR2	Under *in vitro* conditions, a subpopulation of macrophages that expresses CXCL7 attracts more U87-MG cells, compared to macrophages without overexpression.^97^
CXCL8 (IL-8)	CXCR1, CXCR2	Neutralization of the IL-8/CXCLR1/CXCR2 axis with an antibody inhibits motility of LN-18 and U87-MG spheroids *in vitro*.^48^
CXCL10 (IP-10)	CXCR3	U87-MG cells transfected to downregulate CXCR3 exhibit decreased migration in a Transwell assay in comparison to non-downregulated U87-MG cells.^98^ Upregulation of CXCR3 in LN229 results in increased tumor motility *in vitro*.^97^ Both inward and outward motility trapping using CXCL10 is proven in the F98 orthotopic rat model.^25^
CXCL12 (SDF-1)	CXCR4, CXCR7	U87-MG cells preferentially migrate toward CXCL12-containing medium in a Transwell assay.^20^ A CXCR7 antagonist inhibits cell invasion of U251-MG and U373-MG cells in 2 separate migration assays.^99^ U87-MG and primary GBM GSC cells migrate from striatum to the SVZ following a CXCL12 concentration gradient in U87-xenograft mice.^22^ Both 2D and 3D cultures of CXCR4-overexpressing U87-MG showed significant migration toward the CXCL12-loaded sponges *in vitro*. When inoculated into nude rats, one out of 3 tumors showed migration toward a CXCL12-loaded sponge.^21^
CX3CL1 (Fractaline)	CX3CR1	Exogenous CX3CL1 induces a dose-dependent migration of U87MG, T98G, and U251 cells *in vitro*. However, decreased migration was demonstrated in the presence of an anti-CX3CL1-antibody or with an siRNA for CXCL31 in these cell lines, indicating that endogenous CX3CL1 may inhibit tumor cell motility.^100^

#### Galvanotaxis

Electrical stimuli, both endogenous and exogenous, influence HGG migration in a process called galvanotaxis.

Neuronal activity generates endogenous electric signals that can direct tumor migration in preclinical HGG models, via both nonsynaptic ionic currents and synaptic communication.^26^^,^^27^ In glioma-bearing mice, chemogenetic neural activation contralateral to the primary tumor site increased migration across the corpus callosum toward the contralateral hemisphere—resembling outward motility trapping—whereas ipsilateral activation enhanced proliferation without affecting migration.^28^ Exogenous electrical stimulation of the brain, widely used in the treatment of neurological and psychiatric disorders, has also been hypothesized to influence HGG migration.^29^ However, HGG cells respond inconsistently to electrical fields in preclinical studies. Their sensitivity and directionality (anodal or cathodal) vary between cell lines, depend on the electrical stimulation parameters, and are influenced by the TME.^30^ Taken together, the electrical redirection of HGG migration remains unpredictable, hindering the development of galvanotaxis-based motility trapping for now.

In contrast, chemical stimuli pose a more consistent and predictable migratory response and are therefore the primary focus of this review. However, the optimal chemoattractant might differ across HGGs—a complexity that will be discussed next.

### Susceptible Tumor Cells: Tackling Tumor Heterogeneity

Most research on HGG migration focuses on GBM, but these findings may not be generalizable across all HGGs due to substantial inter- and intratumoral heterogeneity. Both the TME and chemokine signaling pathways vary between subtypes and may influence susceptibility to motility trapping. For instance, pontine DMG arises in a TME that contains significantly less chemokines and immune cells compared to GBM.^31^ Fewer competing chemoattractant signals may reduce interference and increase the effectiveness of a motility trap. Alternatively, these tumors may be intrinsically less responsive to chemotaxis and may require higher chemoattractant concentrations to be redirected.

Chemotactic behavior also varies within HGG subtypes. Liu et al highlighted variability in expression of CXCR4 and CXCR7 between patient-derived GBM cell lines, with both receptors overexpressed on slow-cycling populations.^32^ Similarly, Suita et al reported variable CXCR4 levels in 6 GBM primary cell lines, with CXCL12 treatment significantly increasing migration in 5 of them, with variable effect sizes.^33^ Differences in expression levels of CCL5 and its receptor CCR5 are also found between GBM subtypes, with the classical subtype exhibiting the highest expression of both.^34^ Tumor-specific chemotactic responses have also been observed. Three primary DMG cell lines exhibited heterogeneous migratory behavior in response to the same chemoattractants, further indicating that chemokine sensitivity is not uniform within a single subtype.^35^

Intratumoral heterogeneity further complicates chemotactic susceptibility. The IvyGAP data revealed regional differences in chemokine-receptor expression within GBM, for example, CXCR4 enrichment in the pseudopalisading regions and CCR10 overexpression in the infiltrative zone.^36^ Moreover, single-cell and spatial multi-omics now enable more precise characterization of subpopulations within 1 tumor, for example, the distinct transcriptomic signatures between migratory cells and bulk tumor. Hu et al analyzed multiregional biopsies from 68 HGG patients, covering both contrast- and noncontrast-enhancing MRI regions, representing bulk and migratory tumor subpopulations, respectively.^37^ A higher proportion of mutations unique to a single biopsy of a particular tumor was observed in the migratory subpopulation compared to the bulk subpopulation, indicating early evolutionary divergence and distinct neoplastic (and potential chemotactic) behavior.^37^ Similarly, Mathur et al performed a spatial analysis from a whole-tumor perspective using human GBM samples.^38^ Neural and proneural samples were located to the margins, while classical and mesenchymal samples were located closer to the core, indicating distinct gene expression profiles between these regions.^38^

To conclude, recent findings highlight significant intertumoral differences in chemokine signaling, providing rationale to tailor motility traps to HGG subtypes or individual patients. The intratumoral heterogeneity between tumor bulk and invasion subpopulations should also be considered, suggesting the assessment of multiregional biopsies in the design of a personalized motility trap.

### Exploring Suitable Delivery Systems

Local administration of the chemokine is crucial for motility trapping, and the delivery system should meet several requirements including biocompatibility. Most importantly, it should maintain a stable concentration gradient to attract tumor cells, requiring sustained and ideally tunable release kinetics. Imaging compatibility enables *in vivo* monitoring of the trap’s position and degradation, while being distinguishable from tumor recurrence on MRI. Providing structural support for migration and capturing the tumor cells within the trap is an added benefit, favoring implantable over infusion-based techniques. Three implantable local delivery systems for chemokines have been tested and are discussed below: bacterial cellulose, aerogel sponge, and hydrogels.

Bacterial cellulose membranes, widely used in neurosurgery for their hemostatic properties, offer robust biocompatibility. However, when loaded with chemokine, a burst release of more than 90% within the first 4 h is noted *in vitro*,^39^ raising concerns regarding their effectiveness to attract tumor cells in a clinical setting. Moreover, residual bacterial cellulose membranes in the resection cavity might resemble tumor remnants or recurrence on postoperative MRI, further questioning their potential for motility trapping.

Second, an aerogel sponge has been developed for local chemokine delivery in the brain.^21^ This highly porous implant is composed of silk fibroin and hyaluronic acid, is visible on MRI, and degrades approximately 1 week after implantation.^21^  *In vitro* release kinetics were not reported, but migration assays showed increased migration of HGG cells toward the chemokine-loaded sponge, relative to the implant without chemokine. The sponge demonstrated moderate cytotoxicity to both tumor and healthy cells. Subsequently, sponges were implanted into the brains of Fisher rats, and histological analysis after 7 days showed a foreign body reaction and entrapment of the chemokine inside the sponge, presumably hindering its applicability as a suitable delivery system.^21^

Implantable hydrogels are commonly used in (pre)clinical research to deliver local therapies for HGG, primarily chemotherapeutics.^40^ These 3D scaffolds, formed through physical or chemical crosslinking, provide structural support for tumor migration and generally demonstrate good biocompatibility.^41^ Their properties can be modified for controlled release of therapeutics or chemokines over time. While standard MRI typically does not visualize hydrogels, specialized MRI techniques or the incorporation of contrast agents enable monitoring.^42^ Additionally, hydrogels not only attract tumor cells but can also retain them within the scaffold.^43^

Khan et al designed a chemokine-loaded hydrogel for GBM with varying loading concentrations.^43^ Interestingly, the lowest concentration had the highest cumulative release, with 98.5% of the loaded compound released at the end of the experiment, whereas the highest concentration provided the most prolonged release (up to 16 days). Degradation of the gel occurred over 7-15 days.^43^ Similarly, Suita et al developed a CXCL12 liposome-embedded hydrogel and obtained a loading efficiency of 99.8% and a sustained release of the chemokine *in vitro* over a period of 2 weeks.^33^ Upon implanting this gel in the brains of C57/B6 mice, no inflammatory effects were observed on cytokine array analyses and immunohistological stainings.^33^

Implantation-based delivery might not be suitable in cases where adding a large additional volume is undesirable, for example, nonresectable tumors. In these situations, infusion via an intracerebral or intratumoral catheter offers a less space-occupying approach and represents a potential alternative. An example is convection-enhanced delivery (CED), which applies infusion under hydrostatic pressure, thereby enabling repeated delivery and deeper distribution of the infusate into surrounding tissue. It has been used for over 2 decades in patients with a primary brain tumor to administer chemotherapy, liposomes, and viruses, but only within clinical trials.^44^ However, the existing clinical framework for CED would facilitate clinical implementation of motility trapping and combining CED with a subcutaneously implanted pump would allow for outpatient delivery.

In summary, the implantable delivery systems investigated for chemokine release in the brain present distinct advantages and limitations. Hydrogels are particularly promising for their modifiable properties, while CED offers an interesting alternative.

### Influence of the TME

Motility trapping redirects malignant cells by exploiting their intrinsic migratory patterns within the TME. This complex ecosystem actively promotes or hinders migration through mechanical and cellular factors, which may facilitate or limit the success of motility trapping.

The mechanical properties of the TME are largely determined by the extracellular matrix (ECM), comprising interstitial fluid, chemokines, and structural proteins (eg, hyaluronic acid and glycoproteins). These proteins serve both as structural support for migration and as a source of mechanical signals sensed by mechanosensitive receptors on tumor cells.^45^ Experimental studies confirm the stiffness-dependent behavior of HGG, with softer environments stimulating migration. Wang et al showed that both pediatric and adult HGG cell lines exhibited increased migration and proliferation in softer hydrogels.^46^ Another study demonstrated that GBM cells cultured in matrices mimicking tumor border stiffness underwent a metabolic shift that increased migratory behavior, while a stiffer environment promoted proliferation.^47^ Despite being stiffer, white matter is generally preferred for migration over gray matter because its aligned axonal architecture provides strong directional guidance.^6^ Perivascular migration is similarly supported by the aligned structure of blood vessels and further enhanced by CXCL8 secretion from endothelial cells.^48^

The cellular TME, including microglia, tumor-associated macrophages (TAMs), reactive astrocytes, pericytes, oligodendrocytes, and endothelial cells—also guides HGG migration. The myeloid compartment plays a central role in redirecting migration by secreting a complex, chemokine-rich mixture, creating a signaling network that is subtype-specific.^49^ Additionally, these cells secrete MMPs to degrade ECM proteins and create space for tumor invasion.^50^ Reactive astrocytes, in turn, stimulate migration by forming gap junctions with HGG cells, facilitating intercellular communication and enhancing motility.^51^ This dynamic TME is poorly captured by *in vitro* research and is only partially present in severely immunocompromised PDX models. Advances in immunocompetent PDX models and single-cell multi-omic sequencing techniques will allow an in-depth correlation of HGG migration patterns with functional TME states, including therapy-induced shifts in cellular composition and chemokine signaling.^52^

Overall, the TME provides an intricate mixture of endogenous cues that influence tumor migration, and overruling or redirecting these factors will be critical for effective motility trapping. They will need to outcompete the existing cues for invasion and be placed with respect to the white matter anatomy, particularly outward motility traps. For nonresectable supratentorial tumors, redirection toward non-eloquent brain regions via white matter tracts offers a plausible strategy. In pontine DMG, the cerebellum may serve as a viable trapping site, given its established connectivity through white matter tracts, which are frequently exploited in the invasive spread of these tumors.^53^ Beyond trap placement, the complex TME poses a significant challenge in the chemoattractant choice and concentration. Computational modeling is a promising strategy to tackle this, having demonstrated predictive power for local drug release in complex environments.^54^ Applied to motility trapping, models could predict the optimal chemoattractant, concentration, release kinetics of the delivery system, and trap placement with the highest likelihood of successfully attracting tumor cells *in vivo*, thereby accelerating translational research.

## Potential Side Effects

The biological effects of chemokines extend beyond attracting tumor cells and lead to potential side effects of motility trapping.

### Tumor-Associated Side Effects

Several chemokines can stimulate tumor growth,^55^ which is a potential concern when designing a motility trap. For example, a CXCL10-loaded hydrogel resulted in increased Ki-67 expression in F98 glioma-bearing rats, unfortunately without evaluating tumor volumes.^25^ Another study noted a trend toward larger tumor volumes after local CXCL12 administration in U87-MG-xenografted rats.^21^ However, the attraction of tumor cells would always be followed by local therapy in our concept to counteract this possible proliferative effect. Moreover, prioritizing chemokines with a lower proliferative effect further mitigates this risk. Importantly, the selected chemokine should induce directional migration rather than increasing invasiveness. The latter would drive tumor cells further into adjacent tissue, undermining the intended effect of motility trapping. Because the potential to stimulate invasiveness varies among chemokines,^55^ careful chemokine selection is critical to minimize tumor-related side effects.

### Immune-Related Side Effects

Motility trapping sits at the intersection of oncology and immunology and therefore carries the potential for immune-related side effects, which can either be beneficial or adverse.

Microglia, TAMs, and astrocytes express various chemokine receptors, meaning exogenous chemokines could significantly alter the TME. One study evaluating the effect of intratumorally implanted CCL2-, CXCL10-, or CCL11-loaded hydrogels using the immunocompetent F98 glioma rat model reported an increase in GFAP expression after administering all 3 chemokines, suggesting astrocyte activation.^25^ However, microglia and TAMs, considered the main contributors to the immunosuppressive microenvironment in HGG,^56^ were not assessed in this study, leaving local immune activation due to chemokine delivery largely unexplored. Especially in pediatric HGGs, the plasticity and heterogeneous landscape of microglia remain one of the key reasons for immunotherapy failure.^49^ Monitoring potential changes in these cellular populations after chemokine administration will therefore be crucial to fully unlock the potential of motility trapping.

Another consideration is the attraction of peripheral immune cells through chemokine administration. However, chemokine selection could be used to tailor this potential immune response, as different chemokines preferentially recruit specific immune cell populations. For instance, CCL2 preferentially attracts monocytes rather than neutrophils or eosinophils, whereas CCL11 is largely selective for eosinophils, and the CXCL12/CXCR4 axis has little effect on immune cells.^57^^,^^58^ This selectivity can be exploited to prevent or leverage specific immune responses.

Certain populations, for example, regulatory T cells (Treg) and myeloid-derived suppressor cells (MDSCs), suppress antitumoral immunity and limit immunotherapy effectiveness.^59^ CCL2 is overexpressed in the glioma microenvironment and is known to recruit these pro-tumoral cells, dampening immune responses and CAR-T cell activity.^60^ Blocking of the CCL2-CCR4 axis in a murine glioma model increased overall survival with a decrease in the proportion of Tregs and MDSCs.^60^ Therefore, chemokines that recruit pro-tumoral populations should be avoided when designing motility traps.

Conversely, motility trapping also offers an opportunity to attract antitumoral immune cells, potentially synergizing with immunotherapy. For example, CXCL9 activates and increases the infiltration of cytotoxic T lymphocytes but is poorly expressed in the GBM microenvironment. Overexpression of this chemokine using gene therapy increased cytotoxic T cell infiltration and synergized with anti-PD-1 immunotherapy in murine GBM models.^61^ Similarly, an immunostimulatory hydrogel loaded with CXCL10 (to recruit activated T lymphocytes) was developed to induce immunogenic cell death in GBM.^62^ Implantation of the hydrogel after tumor resection of GL261-bearing mice decreased tumor volumes, accompanied by an increase in mature dendritic cells and CD8+ T cell/Tregs ratio and an improvement in overall survival.^62^ Another strategy is engineering CAR-T cells to express receptors matched to the chemokine included in the motility trap. In GBM, where CXCL8 is overexpressed after radiotherapy, CAR-T cells overexpressing CXCL8 receptors CXCR1 and CXCR2 demonstrated enhanced homing and antitumor activity compared to their CXCL8 receptor-lacking counterparts.^63^ This underscores that motility trapping can be strategically designed to engage the immune system. Furthermore, selecting chemokines that act on endothelial cells may further enhance systemic therapies, as activation of CCL2 and CCL4 receptors on endothelial cells increases BBB permeability.^64^^,^^65^

### Neurological Side Effects

Neuronal expression of chemokine receptors poses the risk of off-target neurological effects. Chemotaxis of neurons predominantly occurs during embryonic development, where CXCL12 plays a major role, and disruption of this axis leads to cortical disorganization.^66^ In the postnatal brain, only NSCs remain migratory and are thus susceptible to chemotaxis. NSCs exhibit a natural tropism for HGG, a process regulated through CXCL12 and CCL2, in a dose-dependent manner.^67^ Nestin, an NSC marker, is increased in the tumor-bearing hemisphere in the F98 rat model, and this effect appears to be amplified by implanting CCL2-, CXCL10-, or CCL11-loaded hydrogels.^25^ Incorporating these chemokines in a motility trap could recruit additional NSCs to the tumor site, which has proven to exert antitumoral effects in a glioma mouse model.^68^

Multiple chemokines can modulate neuronal morphology and survival. For instance, CXCL12 supports axonal pathfinding of the perforant pathway while promoting dendritic spine formation and synaptic maturation.^69^^,^^70^ After brain injury in CCL5-knockout mice, CCL5 administration restores neuronal dysfunction, both on a histological and behavioral level, by upregulating genes involved in axon and synapse formation.^71^ Moreover, the chemokines CX3CL1 or CCL5 elicit the release of neurotropic factors, suggesting potential neuroprotective effects when administering these chemokines.^72^^,^^73^

Lastly, chemokines can also regulate synaptic activity in a chemokine- and location-specific manner. For example, CXCL12 increases the excitability of dopaminergic neurons in the substantia nigra, leading to contralateral circling behavior when injected in this region in rats.^74^ Multiple chemokines affect hippocampal neuronal excitability and cognition, as summarized by Sowa et al^75^ Supporting this, intraventricular CCL3 injection, acting through CCR5, decreases hippocampal AMPA-dependent synaptic transmission and impairs memory in mice compared to vehicle-injected animals, an effect reversed by co-injection of a CCR5 inhibitor.^76^ Similarly, ventricular administration of CCL2 impairs learning capacity in mice, while CXCL12 infusion in the ventricle induces feeding disorders via overexpression of orexigenic peptides in the hypothalamus.^75^^,^^77^ Importantly, these studies used intraventricular injection at micromolar concentrations, whereas chemotaxis studies use intraparenchymal delivery of chemokines in the nanomolar range. Nevertheless, potential neurological effects should be considered in future development and will need to be distinguished from the neurocognitive deficits caused by the tumor itself.

### ICP Considerations

Finally, motility trapping can help manage ICP concerns. While an influx of cells could potentially contribute to mass effect or edema, outward motility traps may alleviate ICP in critical regions, for example, the brainstem. This dual effect, redirecting tumor cells while mitigating local ICP raise, represents an important potential advantage, particularly for nonresectable tumors.

## Previously Developed Motility Traps for HGG

### Insights from Extracranial Tumors

The first motility traps were developed for extracranial solid tumors and offer valuable insights for HGG applications. For a comprehensive overview of motility trapping strategies in systemic tumors, we refer to the work of Caballero et al.^78^ Multiple studies demonstrated successful *in vitro* attraction of breast, prostate, colorectal, ovarian, and melanoma cancer cells, mainly using chemokines such as CCL7 and CXCL12.^79–81^ Other signaling molecules have also been tested, with subcutaneously administered erythropoietin and RANKL attracting melanoma and breast cancer cells, respectively.^79^^,^^80^ Galvanotactic attraction has been explored as well, demonstrating consistent anodal migration of murine breast cancer cells *in vitro* compared to random migration without an electric field.^82^ This behavior was consistent with metastatic cell lines from this tumor but unfortunately not repeated in a more complex model.^82^ Although not always applicable for HGG, these insights could inspire us to explore new paths in the design of an HGG motility trap.

Extracranial motility traps capture metastatic tumor cells instead of drawing them back to the original organ and are placed subcutaneously or within the peritoneal cavity—locations inapplicable to HGG. Importantly, only a few incorporate an antitumoral component. An example of the latter is M-Trap, an implantable device to treat ovarian cancer loaded with exosomes derived from cancerous ascites fluid.^83^ Surgical removal of the device containing the captured cells significantly increased survival in mice but failed to meet efficacy and safety endpoints in patients, halting its development.^83^^,^^84^ These findings underscore major translational hurdles and emphasize the need for integrated local therapy to obtain a survival benefit while leaving inward motility trapping unexplored.

### Motility Traps for High-Grade Glioma

Four preclinical studies have explored chemokine-based approaches to redirect HGG migration. Autier et al loaded a bacterial cellulose membrane with conditioned media from glioma-associated stromal cells, known to produce, for example, CCL2 and CCL5.^39^ These membranes successfully attracted F98 glioma cells *in vitro*, but when implanted in F98 tumor-bearing organotypic slices, failed to induce migration. The authors propose local chemokine secretion by brain slices overruling the established gradient or insufficient loading or rapid release as an explanation for the lack of migration, but the *in vivo* efficacy thus remains unknown.^39^

Hydrogels loaded with CCL2, CXCL10, or CCL11 have also been tested.^25^ CCL2 and CXCL10 attracted F98 and U87-MG cells *in vitro*, in contrast to CCL11. Combining the chemokines resulted in less migration compared to CXCL10 and CCL2 alone. In the orthotopic F98 rat model, intratumoral delivery of the 3 single chemokines reduced peritumoral clusters, with CXCL10 eliciting the strongest effect. Interestingly, CCL11 also suppressed peritumoral cluster formation *in vivo*. Inoculation of the gel in the contralateral hemisphere to the tumor, mimicking outward motility trapping, increased the number of peritumoral clusters for all single chemokines, with CCL2 showing the most pronounced effect. Chemokine combinations were consistently less effective than the single agents.^25^

Molina-Peña et al developed porous aerogel sponges loaded with CXCL12 for implantation in GBM resection cavities.^21^  *In vitro*, CXCR4-overexpressing U87-MG showed significant migration toward the sponges. In immunocompetent Fisher rats, the sponges elicited a foreign body reaction, which had resolved after 3 months, and were degraded after 76 days without fibrosis. Nude rats were engrafted with CXCR4-overexpressing U87-MG cells and migration toward the sponge occurred in 1 animal. Placing the sponge at a 1-mm distance from the tumor inoculation site resulted in significant detachment and migration of tumor cells toward the chemokine-loaded sponges. CXCL12 delivery tended to reduce survival and increase tumor volume, but only 3 animals were included per group.^21^

Finally, Suita et al developed a CXCL12 liposome-embedded hydrogel, capable of attracting patient-derived stem cell lines *in vitro*.^33^ Subsequently, PDX mice were implanted with CXCL12-loaded hydrogels, 1 mm above the inoculation site. One week later, light sheet microscopy revealed a significant increase in GBM migration toward the CXCL12-loaded trap, compared to empty liposome-embedded hydrogels.^33^

Interestingly, one group engineered polymer conduits containing aligned nanofiber films to mimic white matter tracts and guide GBM migration.^85^ Nude rats xenografted with U87-MG cells underwent a second surgery to implant the conduit containing either aligned nanofibers, smooth polymer films of equivalent thickness, or nothing. The conduit extended from the inoculation site to the craniotomy opening. Eighteen days later, histological analysis showed tumor migration along the entire 5 mm conduit in all conditions, but with markedly more tumor cells in the aligned nanofiber group. This condition led to a significant reduction in total tumor volume, but the study did not evaluate survival.^85^

**Figure 2.**
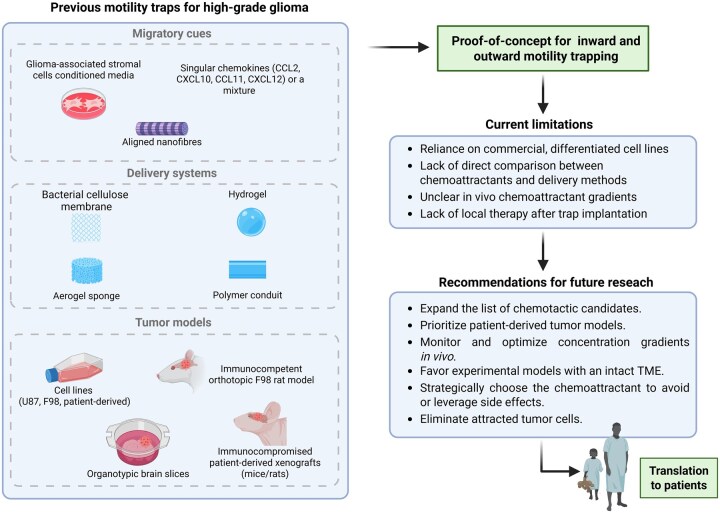
Roadmap illustrating the progression from previous motility traps for high-grade glioma toward clinical implementation (Created in BioRender. Bamelis, A. (2026) https://BioRender.com/1d1qs2a).

Collectively, these studies provide proof-of-concept for both inward and outward motility trapping for HGG, warranting further exploration of this strategy. However, to ensure translatability, important limitations of previous research must be addressed: reliance on CXCL12 and commercial GBM cell lines, lack of in vivo chemoattractant gradient measurement and absence of integrated local therapy. We advocate for more systematic research and outline recommendations for future studies (Figure 2).

## Future Directions

### Recommendation 1: Expand the List of Chemotactic Candidates

Identifying the most effective chemoattractant remains a critical challenge. A wider range of chemoattractants must be tested under standardized experimental conditions to allow direct comparison of their relative strengths. Beyond chemokines, other molecules hold promise for motility trapping. For example, pleiotrophin can modulate HGG migration,^86^^,^^87^ but has not yet been tested for motility trapping. Osteopontin promotes migration by binding to the CD44 receptor on tumors, with *in vitro* studies showing a dose-dependent migratory response in HGG cells.^88^ Exogenous administration of osteopontin in HGG-bearing mice increases the migratory molecule heme oxygenase 1, correlating with more invasive tumor behavior.^88^ The chemoattractants mentioned in this article are not exhaustive and will be further expanded with sequencing techniques.

### Recommendation 2: Prioritize Patient-Derived Tumor Models

Primary cell lines are considered superior to commercial ones for testing therapeutic responses, as they better reflect the genetic and phenotypic features of the parent tumor.^89^^,^^90^ Moreover, they capture tumor heterogeneity to some extent, influencing their responsiveness to a motility trap. Consequently, patient-derived models should be used to assess the effects of redirecting HGG migration, and future research should elucidate whether motility trapping might be applied as a standardized or a personalized approach. If the latter is true, tumor tissue from a biopsy or resection can be used for migration assays in the laboratory to identify the most effective chemoattractant(s) per patient.  This “motilogram” approach aligns with the paradigm shift toward personalized medicine in oncology.^91^

### Recommendation 3: Monitor and Optimize Concentration Gradients In Vivo

Establishing a potent concentration gradient is the cornerstone of motility trapping. Yet the threshold at which a gradient becomes effective and whether a dose-response relationship exists remains unknown. Previous studies did not compare multiple concentrations *in vivo*, and chemokine delivery was rarely measured, leaving the actual exposure to the surrounding tissue unclear. To optimize the concentration gradient, delivery systems with tunable release kinetics are ideal. Additionally, *in vivo* chemotactic gradients should be monitored, for example, through microdialysis, as this strategy is further developed.

### Recommendation 4: Favor Experimental Models With an Intact TME

Previous studies on motility trapping have focused on chemotaxis, without fully accounting for other elements of the TME, possibly explaining the discrepancies observed between *in vitro* and *in vivo* findings. Competing migratory signals from the TME may counteract the administered chemoattractant and a successful motility trap will, therefore, need to overcome these cues. To account for these interfering factors, experimental models should include an intact TME. A suitable *ex vivo* model would be organotypic brain slices *or ex vivo* tissue fragments, as these maintain part of the cytoarchitecture of the brain while allowing serial imaging and cell tracking. Next, animal HGG models should be used to bridge the gap toward the clinic.

### Recommendation 5: Strategically Choose the Chemoattractant to Avoid or Leverage Side Effects

The potential side effects discussed earlier should be closely monitored with serial MRI to evaluate tumor volumes, detailed histological analysis, and behavorial testing for cognitive changes. Because many side effects are immune-related, models with a functional immune system are essential, for example, genetically engineered or humanized mouse models. Once side effect profiles are better characterized, chemoattractants should be selected to minimize harm, have beneficial side effects or synergize with other therapies.

### Recommendation 6: Eliminate Attracted Tumor Cells

Prior work indicates that eliminating the attracted malignant cells is necessary to achieve a survival benefit,^21^^,^^83^ reinforcing the 2-step “attract to kill” approach. Previous research has targeted attracted tumor cells by radiotherapy, resection, or chemotherapy. Moreover, studies should monitor the timeframe in which attraction of tumor cells to the trap occurs, for example, by using fluorescently labeled cells, to determine the optimal point to initiate the second step.

These recommendations aim to accelerate the translational process. Once a functional motility trap for HGG has proven safe and effective in animal models, it will need to be submitted to regulatory agencies before clinical testing. Depending on its design, it may fall under medical device, drug, or combination product regulations. Early engagement with regulatory bodies can optimize trial design and expedite access for patients. Once approved, motility trapping can be integrated into a combined approach, ideally before adjuvant therapy, given its potential to enhance various therapies. By concentrating tumor cells at defined sites, local therapies—surgery, radiotherapy, or emerging strategies (eg, oncolytic viruses)—can target a larger number of tumor cells. However, any surgical procedure of the brain triggers a local inflammatory response, altering chemokine dynamics around the resection cavity. Consequently, effective motility trapping might necessitate a higher chemotactic gradient during the early postoperative period. Practically, if implanted during a GBM resection, it could be integrated into the standard of care, enabling tumor attraction in the first 2 to 3 weeks post-surgery, after which radiotherapy and Temozolomide would commence. Interestingly, other systemic therapies such as chemotherapeutics, targeted agents, and radionuclide therapy may benefit by focusing on the tumor cells in regions where the BBB is compromised or transiently opened via focused ultrasound, thereby maximizing therapy exposure. Beyond these benefits, motility trapping can also prime the immune system, converting immunologically “cold” tumors into targets for both endogenous immunity and immunotherapy.

## Conclusion

Motility trapping transforms the highly migratory behavior of HGG, generally considered a key factor in therapy failure, into a therapeutic opportunity. The 2 strategies, inward and outward motility trapping, each offer advantages depending on the clinical context, and their proof-of-concept has been established in preclinical research. Four elements must be optimized to attract migratory tumor cells and improve (local) therapy effectiveness: (1) an effective migratory stimulus, (2) tumor cell susceptibility, (3) a suitable delivery system, and (4) the iinfluence of the TME. Recent advances in unraveling the TME, developing representative preclinical HGG models, and understanding tumor heterogeneity create momentum to execute the roadmap toward clinical translation proposed here. If successful, motility trapping could synergize with existing treatments and improve outcomes for patients with HGG.

## Data Availability

No new data were generated or analyzed in support of this review article.
